# World Health Organization (WHO) guidelines on use of medically important antimicrobials in food-producing animals

**DOI:** 10.1186/s13756-017-0294-9

**Published:** 2018-01-17

**Authors:** Awa Aidara-Kane, Frederick J. Angulo, John M. Conly, Yuki Minato, Ellen K. Silbergeld, Scott A. McEwen, Peter J. Collignon, for the WHO Guideline Development Group, Hanan Balkhy, Peter Collignon, John Conly, Cindy Friedman, Aidan Hollis, Samuel Kariuki, Hyo-Sun Kwak, Scott McEwen, Gérard Moulin, Antoinette Ngandjio, Bernard Rollin, Flávia Rossi, David Wallinga

**Affiliations:** 1 Department of Food Safety and Zoonoses (NMH/FOS), World Health Organization, 20 Avenue Appia, CH-1211 Geneva 27, Switzerland; 2 Division of Global Health Protection, Center for Global Health, Centers for Disease Control and Prevention, 1600 Clifton Road, MS D-63, Atlanta, GA 30033 USA; 3 Departments of Medicine, Microbiology, Immunology & Infectious Diseases, and Pathology & Laboratory Medicine, Synder Institute for Chronic Diseases and O’Brien Institute for Public Health, Cumming School of Medicine, University of Calgary and Alberta Health Services, Calgary, Canada; 4 Department of Environmental Health, Johns Hopkins Bloomberg School of Public Health, 615 North Wolfe Street, Baltimore, MD 21218 USA; 5 Department of Population Medicine, University of Guelph, Guelph, N1G 2W1 Canada; 6 Infectious Diseases and Microbiology, Canberra Hospital, Canberra, Australia and Medical School, Australian National University, PO Box 11, Woden, ACT 2606 Australia

**Keywords:** Antimicrobial resistance, Antimicrobial use, Agriculture, Food safety, Zoonoses, Health consequences, Guidelines

## Abstract

**Background:**

Antimicrobial use in food-producing animals selects for antimicrobial resistance that can be transmitted to humans via food or other transmission routes. The World Health Organization (WHO) in 2005 ranked the medical importance of antimicrobials used in humans. In late 2017, to preserve the effectiveness of medically important antimicrobials for humans, WHO released guidelines on use of antimicrobials in food-producing animals that incorporated the latest WHO rankings.

**Methods:**

WHO commissioned systematic reviews and literature reviews, and convened a Guideline Development Group (GDG) of external experts free of unacceptable conflicts-of-interest. The GDG assessed the evidence using the Grading of Recommendations Assessment, Development and Evaluation (GRADE) approach, and formulated recommendations using a structured evidence-to-decision approach that considered the balance of benefits and harms, feasibility, resource implications, and impact on equity. The resulting guidelines were peer-reviewed by an independent External Review Group and approved by the WHO Guidelines Review Committee.

**Results:**

These guidelines recommend reductions in the overall use of medically important antimicrobials in food-producing animals, including complete restriction of use of antimicrobials for growth promotion and for disease prevention (i.e., in healthy animals considered at risk of infection). These guidelines also recommend that antimicrobials identified as critically important for humans not be used in food-producing animals for treatment or disease control unless susceptibility testing demonstrates the drug to be the only treatment option.

**Conclusions:**

To preserve the effectiveness of medically important antimicrobials, veterinarians, farmers, regulatory agencies, and all other stakeholders are urged to adopt these recommendations and work towards implementation of these guidelines.

## Background

The increasing prevalence of antimicrobial resistance is a worldwide problem [1, 2]. Antimicrobial-resistant bacteria cause infections that are more severe and less responsive to treatment. Compared with infections caused by susceptible bacteria, infections caused by antimicrobial-resistant bacteria result in increased frequency of hospitalization, prolonged hospitalization, increased duration of illness, and increased mortality [3, 4]. Many factors contribute to increasing antimicrobial resistance in human pathogens including antimicrobial use in humans and in food-producing animals [5, 6].

To address the growing public health problem of antimicrobial resistance, the 68th World Health Assembly adopted the World Health Organization’s (WHO) Global Action Plan on Antimicrobial Resistance (GAP) in May 2015 [7]. The WHO GAP aims to control antimicrobial resistance through various interventions including reducing medically unnecessary use of antimicrobials in humans and in animals [8]. The plan also emphasizes the need for a “One Health” approach for control of antimicrobial resistance with contributions from many disciplines including human medicine and veterinary medicine. Recognizing the inter-disciplinary need to address antimicrobial resistance, the assemblies of the Food and Agriculture Organization of the United Nations (FAO) and World Organisation for Animal Health (OIE) adopted resolutions supporting the WHO GAP in 2015 [9, 10].

Antimicrobials are widely used in food-producing animals for therapeutic use (treatment of ill animals), prophylactic use (disease prevention in healthy animals considered at risk of infection), and growth promotion use (to improve rate of gain or feed efficiency) [11]. Such usage can result in selection and dissemination of antimicrobial-resistant bacteria in food-producing animals which can be transmitted to humans via food and other transmission routes [12, 13]. In 2005, WHO organized an expert committee to classify the importance of antimicrobials used in humans (i.e., medically important antimicrobials) for the purpose of mitigating risks of adverse human health consequences due to use of antimicrobials in food-producing animals [14]. The committee developed criteria to classify these antimicrobials as either important, highly important, or critically important for human medicine, resulting in the WHO List of Critically Important Antimicrobials for Human Medicine (WHO CIA List); the fifth revision of the WHO CIA List was published by WHO in 2016 [15].

With the ever growing public health threat of antimicrobial resistance and the recognized need to preserve the effectiveness of medically important antimicrobials, particularly those antimicrobials judged to be critically important to human medicine on the WHO CIA List, WHO elected to develop formal, evidence-based, guidelines on use of antimicrobials in food producing animals.

## Methods

### Guideline development process (Table 1)

In accordance with the *WHO Handbook for Guideline Development*, three groups were organized [16]. A WHO Steering Group, comprised of nine WHO staff members and a representative each from FAO and OIE, guided the development process. The WHO staff were from five WHO regional offices and from WHO headquarters programs involved with four of the “work streams” of the WHO GAP (Rational Use, National Action Plans and Surveillance, Infection Prevention and Control, and One Health). The FAO and OIE representatives provided comments on the guidelines, but were neither responsible for, nor were asked to endorse, the contents of the guidelines.

**Table 1:** Summary of the WHO guideline development process

Stage	Required steps to develop WHO Guidelines
Planning	WHO Technical Unit
	• Form the WHO Steering Group (WSG)
	WSG
	• Draft the scope of the guideline; begin preparing the planning proposal
	• Identify potential members of the GDG and its Chair(s)
	• Obtain declaration of interests and manage any conflicts of interest among potential GDG members
	WSG and GDG
	• Formulate key questions in PICO format; prioritize outcomes
	WSG
	• Finalize the planning proposal and submit it to the GRC for review
	GRC
	• Review and approve the planning proposal
Development	Commissioning of systematic reviews
	• Develop a process to commission systematic reviews through a request for proposals
	• Perform systematic reviews of the evidence for each key question, and any additional literature reviews as needed
	• Evaluate the quality of the evidence for each important outcome, using GRADE methodology
	WSG
	• Convene meetings among the GDG members and review
	GDG
	• Formulate recommendations using the GRADE framework
	External review group
	• Conduct external peer review
Publishing and updating	WSG
	• Finalize the guideline document; perform copy-editing and technical editing; submit the final guideline to the GRC for review and approval
	GRC
	• Review and approve the final guideline
	WSG
	• Finalize the layout; proofread
	• Publish, disseminate, implement
	WHO Technical Unit
	• Evaluate and update

*WHO* World Health Organization, *WSG* WHO Steering Group, *GDG* Guideline Development Group, *GRADE* Grading of Recommendations Assessment, Development and Evaluation, *GRC* Guidelines Review Committee, *PICO* population, intervention, comparator, and outcome

A Guideline Development Group (GDG), comprised of thirteen external experts from five WHO regions with expertise in clinical human medicine, infection prevention and control, evidence-based medicine, tropical medicine, epidemiology, veterinary medicine, microbiology, infectious diseases, public health, antimicrobial resistance, economics and veterinary ethics. The GDG assessed the available evidence and formulated recommendations. One member of the GDG was a Grading of Recommendations Assessment, Development and Evaluation (GRADE) methodologist. GDG members were selected in a way that sought geographic representation and gender balance, and avoided conflicts of interest. An External Review Group (ERG), composed of eleven technical experts and stakeholders with interest in use of antimicrobials in food-producing animals including veterinarians, microbiologists, animal scientists, public health practitioners, food safety experts, public health policy officials, and physicians. The ERG reviewed the final draft of the guidelines to identify errors of fact and commented on the clarity of the language, contextual issues and implications for implementation.

### Retrieval of evidence

WHO commissioned systematic reviews of the published evidence that restrictions in use of antimicrobials in food-producing animals reduced the prevalence of antimicrobial resistance in bacteria isolated from food-producing animals and humans (the specific PICOT [P = population, I = intervention, C = comparator, O = outcome, T = time] formatted questions used to direct the systematic reviews are available in the WHO guidelines on the WHO website at http://apps.who.int/iris/bitstream/10665/258970/1/9789241550130-eng.pdf). Following a public solicitation for applications, the WHO Steering Group selected the applications from teams at Bond University (Australia) and University of Calgary (Canada). The systematic review teams interacted regularly with the WHO Steering Group but worked independently of each other.

WHO also commissioned narrative content specific literature reviews on [1] illustrative examples of probable transfer of resistance determinants from food-producing animals to humans, [2] biological plausibility of associations between antimicrobial use in food-producing animal production and increased risks of human exposures to and infection by antimicrobial resistant zoonotic pathogens, and [3] unintended consequences associated with restrictions on antimicrobial use in food-producing animals.

### Assessment of the quality of the evidence

Using procedures described in the *WHO Handbook for Guideline Development* [16], the GDG made a final judgment of the quality of the evidence, after considering the limitations, inconsistency, indirectness, imprecision, publication bias of the studies, and any upgrading criteria for each of the relevant outcomes [17].

### Formulation of recommendations

The commissioned reviews were presented to the GDG at in-person meetings in October 2016 (Raleigh, North Carolina) and March 2017 (Geneva, Switzerland). The GDG formulated recommendations based upon the summarized evidence and judgments of the overall quality of the evidence. Using the GRADE approach, the GDG also judged the strength of each recommendation by considering the importance of the problem, the quality of evidence, the balance between benefits and harms, the values and preferences of affected populations, resource implications, the impact on equity, human rights, gender and social determinants of health, acceptability, and feasibility [17]. In accordance with the *WHO Handbook for Guideline Development* [16], the GDG classified a recommendation as strong when there was “confidence that the desirable effects of adherence to the recommendation clearly outweigh the undesirable effects.” The GDG also classified a recommendation as conditional when “the quality of the evidence supporting the recommendation is very low, or the recommendation may apply only to specific groups or settings.”

### Declaration of interests by external experts and contributors

As per WHO regulations, all external experts and contributors disclosed their secondary interests (e.g., financial and intellectual) prior to participating in the WHO guideline development process. When a conflict of interest was identified, WHO followed standard procedures to determine whether or not the expert was permitted to participate in the guideline development process. A biographical sketch of each nominated GDG member was posted online for public comments. The WHO Steering Group reviewed all information gathered before approving final GDG membership.

### Role of the funding source

Governments of Japan and The Netherlands provided funding for the guidelines but were not involved with guidelines development. WHO facilitated the development of the WHO Guidelines, in accordance with the WHO guideline development process, and provided additional funding for the guidelines.

## Results

### Evidence

The systematic reviews yielded a large number of studies that demonstrated a consistent decrease in the prevalence of antimicrobial resistance in bacteria isolated from food-producing animals or humans following restrictions on use of medically important antimicrobials in food-producing animals. The Bond University systematic review team provided a narrative report of the quality of, and summary of findings from, 93 studies and concluded that limiting the antimicrobial use in food animals is likely to reduce the presence of antimicrobial resistance in food –producing animals and humans. The University of Calgary systematic review team provided a quantitative report from 177 studies that included an assessment of the quality of the primary studies and provided a meta-analysis of the risk differences for reductions in the prevalence in antimicrobial resistance reported with various restrictions on antimicrobial use in food producing animals [18]. The University of Calgary team used the quality assessment and pooled data to generate GRADE evidence profiles and concluded that there is a large body of evidence that, when pooled, consistently shows that restrictions on the use of antimicrobials in food-producing animals are associated with reductions in the presence of antimicrobial-resistant bacteria in these animals and humans, particularly humans in direct contact with these animals. Full reports of the systematic reviews are available on the WHO website at http://apps.who.int/iris/bitstream/10665/259241/1/WHO-NMH-FOS-FZD-17.2-eng.pdf.

Of the three commissioned content specific literature reviews, one review provided evidence from illustrative examples of probable transfer of resistant determinants for streptothricins, glycopeptides and colistin from food-producing animals to humans [19]. The second review concluded that there was a large amount of mechanistic information on antimicrobial resistance that indicates that use of antimicrobials in food-producing animals selects for antimicrobial resistance in bacteria in food-producing animals that disseminates among food-producing animals, their environment, and to humans. The third review found that unintentional consequences of restrictions on antimicrobial use in food-producing animals (e.g. adverse effects on animal health and welfare, food safety, productivity, economic outcomes) were minor and temporary. The findings of the latter two reviews are available on the WHO website at http://apps.who.int/iris/bitstream/10665/259241/1/WHO-NMH-FOS-FZD-17.2-eng.pdf .

### Quality of evidence

The evidence presented to the GDG was predominately from observational and ecologic studies. Additionally, the systematic reviews found few studies from middle-income and low-income countries, and few studies included small-scale food-producing animal operations. Despite these limitations, the GDG determined that there was sufficient evidence that use of medically important antimicrobials in food-producing animals selects for antimicrobial resistance in bacteria, and that such antimicrobial resistant bacteria can be transmitted to humans, including in middle-income and low-income countries, and small-scale operations. Summaries of the information used by the GDG to develop best practice statements and recommendations related to the use of medically important antimicrobials in food-producing animals, including evidence profiles, summary of findings tables, and evidence-to-recommendations tables, are available at http://apps.who.int/iris/bitstream/10665/259242/1/WHO-NMH-FOS-FZD-17.3-eng.pdf .

### Best practice statements (Table 2)

The GDG developed two best practice statements for classes of antimicrobials not currently used in food-producing animals.

1. Any new class of antimicrobials or new antimicrobial combination developed for use in humans will be considered critically important for human medicine unless categorized otherwise by WHO.
2. Medically important antimicrobials which are not currently used in food production should not be used in the future in food production, including in food-producing animals or plants.

**Table 2:** Best practice statements on use of medically important antimicrobials in food-producing animals

Best practice statements	
1	Any new class of antimicrobials or new antimicrobial combination developed for use in humans will be considered critically important for human medicine unless categorized otherwise by WHO.
2	Medically important antimicrobials that are not currently used in food production should not be used in the future in food production including in food-producing animals or plants.*
	*Although these guidelines only pertain to use of medically important antimicrobials in food-producing animals, the GDG concluded that this best practice statement ought to apply to all antimicrobial uses in food-producing animals and in plants. All such uses have the potential to select for antimicrobial resistance, which can be subsequently transferred to humans.

The GDG judged that these best practice statements were needed to provide a basis for taking actions to preserve the effectiveness of critically important antimicrobials for humans (including some that are the only antimicrobial treatment option for seriously ill persons) that are not currently used in food-producing animals. Furthermore, given the critical need for new classes of antimicrobials for the treatment of some serious and life-threatening infections in humans, the development and eventual marketing of new classes of antimicrobials will likely be antimicrobials required for infections in humans with no or limited treatment options.

Since these antimicrobials are not yet used in food-producing animals, direct evidence (e.g. derived from epidemiological studies of effects of restrictions on use of the antimicrobials in food-producing animals on antimicrobial resistance outcomes) is not available. Instead, the best practice statements are supported by considerable indirect evidence, including a large body of evidence from the systematic reviews and from mechanistic studies of antimicrobial resistance [18, 19].

### Recommendations (Table 3)

The GDG developed four recommendations, supported by both direct and indirect evidence, concerning the use of medically important antimicrobials in food-producing animals.

1. An overall reduction of use of all classes of medically important antimicrobials in food-producing animals is recommended. (Strong recommendation, low-quality evidence).
2. Complete restriction of use of all classes of medically important antimicrobials in food-producing animals for growth promotion is recommended. (Strong recommendation, low-quality evidence).
3. Complete restriction of use of all classes of medically important antimicrobials in food-producing animals for prevention of infectious diseases that have not yet been clinically diagnosed is recommended. (Strong recommendation, low-quality evidence).
4. It is suggested that antimicrobials classified as highest-priority critically important for human medicine should not be used for treatment of food-producing animals with clinically diagnosed infectious disease. It is also suggested that antimicrobials classified as critically important for human medicine should not be used for control of the dissemination of a clinically diagnosed infectious disease identified within a group of food-producing animals. (Conditional recommendation, very low-quality evidence).

**Table 3:** Recommendations on the use of medically important antimicrobials in food-producing animals

Recommendations	
1	The GDG recommends an overall reduction in use of all classes of medically important antimicrobials in food-producing animals.
2	The GDG recommends complete restriction of use of all classes of medically important antimicrobials in food-producing animals for growth promotion.
3	The GDG recommends complete restriction of use of all classes of medically important antimicrobials in food-producing animals for prevention of infectious diseases that have not yet been clinically diagnosed.
	*Specific considerations: when a veterinary professional judges that there is a high risk of spread of a particular infectious disease, use of antimicrobials for disease prevention is justified, if such a judgement is made on the basis of recent culture and sensitivity testing results.*
4	a – The GDG suggests that antimicrobials classified as critically important for human medicine should not be used for control of the dissemination of a clinically diagnosed infectious disease identified within a group of food-producing animals. b – The GDG suggests that antimicrobials classified as highest priority critically important for human medicine should not be used for treatment of food-producing animals with a clinically diagnosed infectious disease.
	*To prevent harm to animal health and welfare, exceptions to recommendations 4a and 4b can be made when, in the judgment of veterinary professionals, bacterial culture and sensitivity results demonstrate that the selected drug is the only treatment option.*

The GDG determined that the first three recommendations should be strong despite the low-quality evidence due to the large human health benefits of lowered prevalence of antimicrobial resistance in bacteria isolated from humans and the limited evidence of undesirable consequences of restrictions on antimicrobial use of medically important antimicrobials in food producing animals. Specifically, there was a large amount of consistent evidence from the systematic reviews and the literature reviews that resistant bacteria can spread among food-producing animals, into their environment, and to humans, and that restrictions on use of antimicrobials in food-producing animals reduces the prevalence of antimicrobial resistance in bacteria isolated from food-producing animals that are, and can be, transmitted to humans. The GDG also concluded that for the fourth recommendation, although evidence from the systematic reviews and additional studies indicates it will achieve the human health benefit of lowered antimicrobial resistance in bacteria, this recommendation should be conditional due to the very low quality of available evidence. In order to protect animal health and welfare, the GDG concluded that exceptions to recommendations 4a and 4b can be made when, in the judgement of qualified veterinary professionals, susceptibility results demonstrate that the selected drug is the only treatment option.

The GDG also concluded that in order for people in all countries to realize the benefits of continued effectiveness of medically important antimicrobials, the recommendations in these guidelines should be applied in all animal production settings and in all countries.

#### Further research

With the recognition of the need to reduce the use of medically important antimicrobials in food-producing animals, the GDG identified the following research areas which can be addressed to help facilitate reduced antimicrobial use: [1] identification of the most effective methods for implementing antimicrobial stewardship programs in food-producing animals, and better understanding of values and preferences of those affected by these programs [2] cost-effectiveness studies of interventions aimed at reducing antimicrobial use in food-producing animals [3] effects of restriction of antimicrobial use for disease control and treatment in food-producing animals on antimicrobial resistance in bacteria isolated from animals and humans [4] development of rapid diagnostic and antimicrobial sensitivity tests, and [5] effects of restriction of antimicrobial use in food-producing animals in low- and middle-income countries on antimicrobial resistance in bacteria isolated from animals and humans, and on unintended consequences.

## Discussion

The use of antimicrobials in food-producing animals has been a public health concern for decades [20]. In 1997, a WHO consultation concluded that use of antimicrobials in food-producing animals selects for resistant bacteria, and such resistant bacteria are transmitted to humans in food and through direct contact with animals [21]. WHO has attempted to mitigate this public health threat for many years. In 2000, WHO, with participation of FAO and OIE, developed Global Principles for Containment of Antimicrobial Resistance in Animals Intended for Food [22]. Recommendations from these and other WHO consultations provided a framework for several countries [23, 24], and some food companies [25], to restrict selected uses of antimicrobials in food-producing animals. Nevertheless, WHO determined that formal guidelines, supported by available evidence, were needed to advance the WHO GAP to control antimicrobial resistance.

In accordance with the WHO guideline development process [16], the GRADE approach was used to assess the quality of the evidence used to formulate recommendations for these guidelines.

The GRADE approach follows a rigorous and transparent process, and is the most widely used approach for evidence appraisal for guideline development. The GRADE approach was initially developed for decision-making in clinical settings, where randomized clinical trials are commonly available. It rates evidence derived from randomized controlled trials as high quality, while evidence from epidemiological studies, surveillance programmes and other sources of observational data, in which confounding cannot be adequately controlled, is rated as “low quality”. In common with many important public health issues, due to ethical and technical complexity, there are no randomized controlled studies that have assessed associations between use of antibiotics in food-producing animals and risks of human exposure to infection by drug-resistant zoonotic pathogens. Most of the supporting scientific evidence, representing the best available evidence, was collected from epidemiological studies, surveillance programmes and observational studies. In addition to the quality of the evidence and the balance of benefits and harms of an intervention, a number of other factors are considered when GDGs formulate recommendations. These factors include the relative value placed on the potential beneficial and harmful outcomes of an intervention, the acceptability and feasibility of the intervention, resource considerations, and effects on equity across subpopulations. A “strong recommendation” means that after taking all relevant considerations into account, the GDG is reasonably certain that the desirable consequences (benefits) of an intervention outweigh the undesirable consequences (risks or harms), such that the intervention should be implemented in most settings.

The GRADE approach has been used successfully in public health settings where only observational studies are available. There are challenges, however, in using the GRADE approach in all settings, especially in the formulation of recommendations on complex interventions in public health settings [26]. Although alternative approaches to GRADE have been suggested [27], WHO continues to rely on the GRADE approach as the most appropriate method for evidence appraisal for guideline development. Importantly, it has been acknowledged that the implementation in 2007 at WHO of a guideline development process using the GRADE approach has resulted in important improvements in the quality of WHO guidelines [28].

Using the rigorous and transparent WHO guideline development process, the GDG determined that three of the four recommendations on use of medically important antimicrobials in food-producing animals should be strong recommendations given (1) the large body of evidence from the commissioned systematic reviews using a rigorous and evidence based approach of the strength and consistency of the association between restrictions of antimicrobial use in food-producing animals and antimicrobial resistance in animals and humans, (2) the suggestion of a dose-response relationship with this association, (3) the large potential health benefits, and (4) the evidence of limited unintended consequences. The GDG also recommended implementation of the WHO guidelines worldwide. As with all WHO guidelines related to food safety, implementation relies on the impetus of Member States and other stakeholders, including the FAO/WHO Codex Alimentarius Commission, to translate the recommendations into national and international standards or guidance. In order to reach the overall goal of reducing antimicrobials in food-producing animals, there is a particular need to identify management alternatives for use of antimicrobials for disease prevention. The GDG acknowledged that implementation in lower-income or middle-income countries may require special considerations such as assistance in improvement of hygienic and agricultural practices including improved housing and husbandry. Furthermore, many countries may need technical and laboratory capacity building assistance for conducting the recommended bacterial culture and antimicrobial susceptibility testing. It is important for countries to conduct surveillance and monitoring of antimicrobial usage in food-producing animals to evaluate the effectiveness of any interventions.

## Conclusion

In conclusion, the WHO guidelines recommend overall reduced use of medically important antimicrobials in food-producing animals, including complete restriction of uses of medically important antimicrobials for growth promotion and for disease prevention. Exceptions for disease prevention can be accepted when based upon the judgement of a veterinary professional and if such judgement is based on recent culture and sensitivity testing results. The WHO Guidelines also suggested that antimicrobials identified as critically important for human medicine not be used in food-producing animals for treatment or disease control unless susceptibility testing demonstrates the drug to be the only treatment option. To preserve the effectiveness of medically important antimicrobials, all stakeholders should implement the recommendations in the WHO guidelines. Such implementation will support the WHO GAP to control antimicrobial resistance by reducing unnecessary use of antimicrobials in food-producing animals.
